# Feature-based interaction between masks and target in continuous flash suppression

**DOI:** 10.1038/s41598-023-31659-9

**Published:** 2023-03-22

**Authors:** Jan Drewes, Christoph Witzel, Weina Zhu

**Affiliations:** 1 Institute of Brain and Psychological Sciences, Sichuan Normal University, Chengdu, China; 2 School of Psychology, University of Southampton, Southampton, UK; 3 School of Information Science, Yunnan University, Kunming, China

**Keywords:** Visual system, Perception

## Abstract

Continuous flash suppression (CFS) has become one of the most popular tools in the study of visual processing in the absence of conscious awareness. Studies use different kinds of masks, like colorful Mondrians or random noise. Even though the use of CFS is widespread, little is known about some of the underlying neuronal mechanisms, such as the interactions between masks and stimuli. We designed a b-CFS experiment with feature-reduced targets and masks in order to investigate possible effects of feature-similarity or -orthogonality between masks and targets. Masks were pink noise patterns filtered with an orientation band pass to generate a strong directionality. Target stimuli were Gabors varying systematically in their orientational alignment with the masks. We found that stimuli whose orientational alignment was more similar to that of the masks are suppressed significantly longer. This feature-similarity (here: orientation) based enhancement of suppression duration can be overcome by feature orthogonality in another feature dimension (here: color). We conclude that mask–target interactions exist in continuous flash suppression, and the human visual system can use orthogonality within a feature dimension or across feature dimensions to facilitate the breaking of the CFS.

## Introduction

One of the most important questions in cognitive neuroscience is whether and how sensory stimuli are processed within and outside of conscious awareness (e.g.^1–4^). In the last several years, the results from studies employing a technique known as continuous flash suppression (CFS)^5^ have been used as one of the main sources of evidence that stimuli that do not reach conscious awareness—and are therefore considered to be subjectively “invisible”—may still be processed to some degree by the visual system (for a review, see^2^).

Commonly, in a CFS paradigm, masks are flashed in a continuing fashion to only one eye. This causes a static image presented only to the other eye to be suppressed from perceptual awareness^5,6^. This un-aware sensation/perception of the static image typically lasts for a period of time significantly longer than what would be expected from conventional binocular rivalry. The paradigm/technique has originally been introduced in the context of afterimages^5^, and by now it has been used to investigate and reveal/show unconscious (or unaware) processing of a number of low-level visual features. These include orientation^6,7^, motion^8^, spatial information^9^, and unconscious binding of low-level visual features based on Gestalt grouping cues, such as good continuation and proximity^10^. Even some effects commonly attributed to “high-level” visual processing stages have been reported to occur without the observer being aware of the percept, for example in face inversion^11–13^, facial expressions^14,15^, semantic information processing^11,16,17^, and information integration^18–20^. Probably due to its power and versatility, in recent years CFS has become widely used to investigate visual processing outside of (subjective) conscious awareness. Still, the exact nature of the underlying neuronal mechanisms is not well understood^2,21–27^.

In most of the relevant studies, the suppression masks were highly salient and colorful, frequently designed from overlapping rectangles and other geometric shapes of different color or luminance. Being somehow similar to Mondrian’s paintings, these masks have been dubbed “Mondrians”. However, a large number of studies utilizing CFS neither specified in sufficient (or even any) detail how their respective masks were designed nor explain why Mondrians rather than other possible masks were chosen in their research. Recent studies found that CFS effectiveness is influenced by both temporal and spatial factors^21,27,28^. In terms of spatial features, mask density (the average size and number of patches per mask) and, in consequence, spectral density, have been shown to affect suppression effectiveness^21^. The duration of suppression was negatively affected when reducing the number of edges in the masks. Further investigation of different kinds of random noise for CFS masking found that spatial pink noise (1/F noise) achieved the longest suppression duration compared to other random noises^29^. Other features and specially constructed masks have also received some recent attention^30–32^. Contrary to this, the direct relationships between masks and targets have received very little if any attention, even though in binocular rivalry^33,34^ interactions in suppression (or dominance) duration between rivaling stimuli are well documented^35–39^. However, the properties of binocular rivalry do not translate immediately to continuous flash suppression^29^. It is therefore unclear whether CFS suppresses a given target globally or if the suppression depends on an interaction between the target and the masks, the degree of which may then be dependent on the similarity of the features contained in both masks and target. If such a feature-based mask-target interaction exists, the suppression depth achieved with a certain kind of mask may then not be the same for all kinds of targets. Targets with features that are similar to the masks may need longer to reach awareness than those with dissimilar features.

To investigate this question, we created sets of targets and masks that vary only in a very specific feature, allowing us to systematically test the effects of feature similarity between masks and targets. We chose spatial orientation as well as color as our features of interest, as these can be controlled and manipulated independently of each other.

## Experiment 1: orientation

In binocular rivalry, interactions between mask and target are well known^35–39^. Naturally, in binocular rivalry, masks are generally as static as the target, leading to an also static relationship between the two. In CFS however at least the masks are highly dynamic; in most CFS experiments, the target is less so, but even a simple contrast ramp may add a dynamic aspect to it. It is therefore not immediately clear if interactions between mask and target similar to those known from binocular rivalry also exist in CFS: at the very least, the dynamic nature of the masks should prevent localized, static (“pixel-by-pixel”) similarities between mask and target from dominating the perceptual relationship between the two. To investigate if and to what extent interactions between mask and target stimuli exist in CFS, as a first step we designed an experiment in which we could gradually control the similarity of one specific feature between masks and targets. The orientation of a Gabor stimulus (a sinusoidal pattern inside a Gaussian envelope) is well suited for this purpose, as the overall structure of the stimulus is simple and the orientation can readily be adjusted to the finest degree possible on a given display system^37^. Unlike previous research in conventional binocular rivalry settings, we used random noise-based masks rather than sinusoidal patterns. Pink noise (1/F frequency characteristics) stimuli have been shown to be an optimal noise for CFS^29^, however they do not have an orientational preference and can therefore not be set in relative alignment to the target orientation. We thus modified pink noise stimuli by applying an orientation bandpass to the noise characteristics, allowing for a strong directional tuning of the noise while otherwise maintaining the randomness of the appearance (see “Methods” section below). While orthogonality in spatial orientation between noise patches has been shown to affect suppression duration^40^, it is not clear to what degree absolute orientation as well as the orientational tuning between masks and target will affect the suppression depth. Research in conventional binocular rivalry would suggest a non-linear relationship with suppression duration peaking when the orientations of mask and stimuli are co-aligned^37^, however to our knowledge no such study exists using CFS as a methodology. Furthermore, most previous research is focused on cardinal orientations only^37,40^. To confirm how much suppression still exists at different degrees of similarity between mask and target, we also added a no-masking baseline for comparison.

### Methods

#### Apparatus

We presented visual stimulation on a 21-inch SUN CRT monitor (1024 × 768 pixel, 120 Hz frame rate with 8 bit resolution per color channel). Participants’ heads were stabilized by a chin-and-head rest, while viewing the presentation through a mirror stereoscope. The physical viewing distance was 56 cm, while the optical distance was increased by approx. 8 cm due to the detour through the mirror stereoscope. The spatial distance between the left and right presentation areas as well as the angle of the mirrors were adjusted for each observer to achieve good fusion of the display. Visual stimuli were presented in Octave 4.2.2^41^ using the Psychophysics Toolbox^42,43^.

#### Stimuli

**Figure 1:**
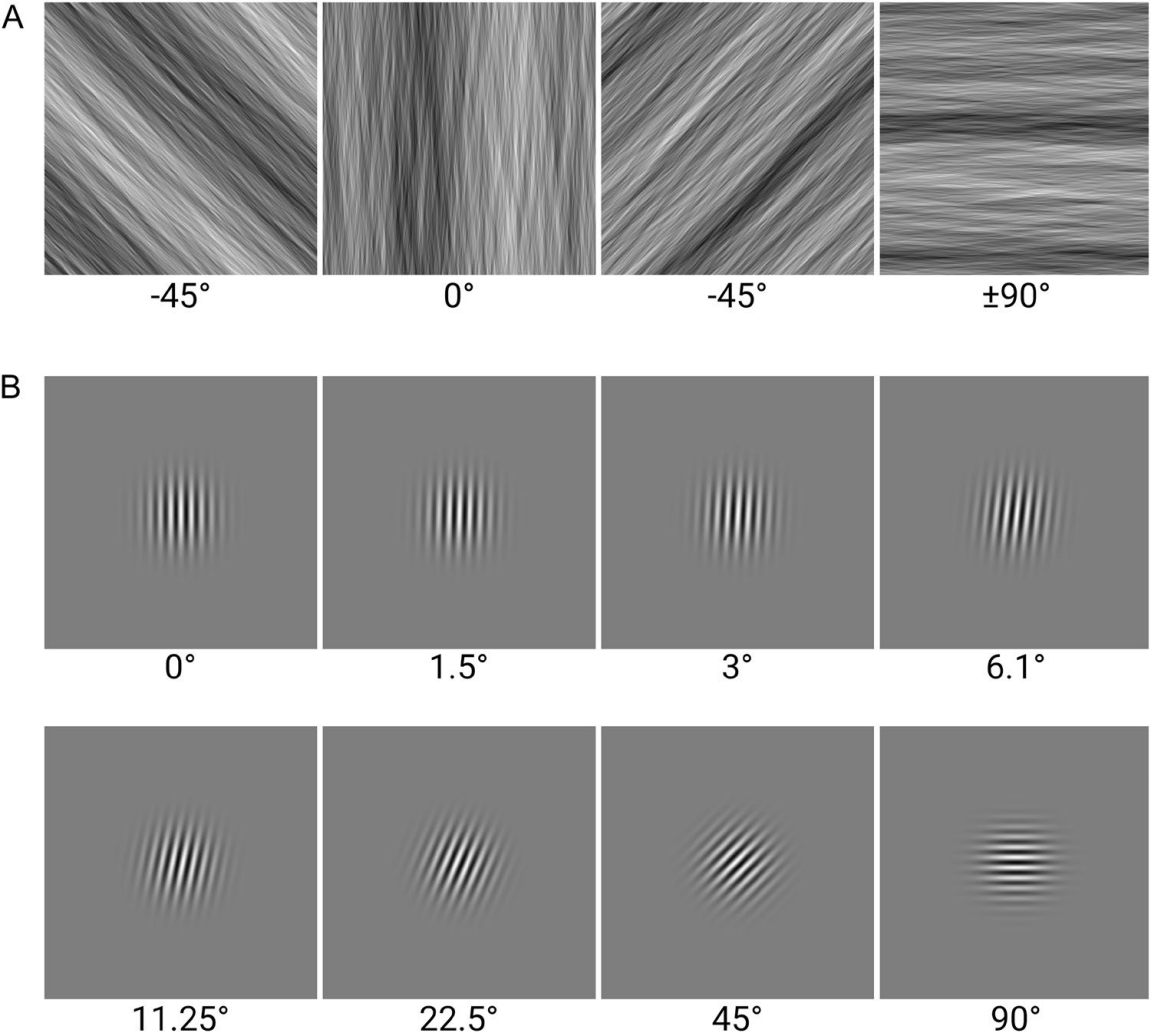
Stimuli used in Experiment 1. (**A**) Sample of mask stimu1i: Pink noise (1/f) filtered with orientation Bandpass (8° fwhh), in the four orientations used. (**B**) Sample of target stimuli: Gabor patches, oriented relative to the vertical (0°) masks (only clockwise orientation shown).

Leaning on the results of our previous study^29^, we created a set of pink noise (1/f) images that were then filtered with an orientation bandpass (see Fig. 1A for examples). The orientation bandpass was applied in Fourier space by means of a Gaussian-shaped orientation-dependent attenuation of the amplitude spectrum: along the preferred orientation the filter value was one, and the further orientations in the spectrum deviated from the preferred orientation the more they were diminished. The filter profile was chosen to be rather narrow (8° fwhh) in order to limit the orientational variability of the masks while still being wide enough to allow for sufficient randomness in the noise pattern. The masks were characteristically defined by the center orientation of this Gaussian bandpass filter. Mask images were square with an edge length of 14.8°. Gray-level Gabor stimuli with a spatial frequency of 4 cycles per degree and a Gaussian envelope of 2.4° fwhh were used as targets (see Fig. 1B), allowing for a controlled adjustment of the degree of co-alignment (similarity) between the orientational content of both masks and targets. Masks and targets were both normalized to a Michelson contrast of 1. Masks were created in 4 Orientations (0°, 45°, 90°, 135° with 0° referring to a vertical main orientation) with 200 samples each. Targets were created in 15 angles of deviation (0 plus 7 steps each for clockwise and counterclockwise: 0°, 1.5°, 3°, 6.1°, 11.25°, 22.5°, 45°, 90°) relative to each of four 4 mask orientations. In this way, the co-alignment between mask and target ranged from perfectly co-aligned to orthogonal. Values were spaced in log-scale with the highest density around the point of alignment.

#### Procedure

**Figure 2:**
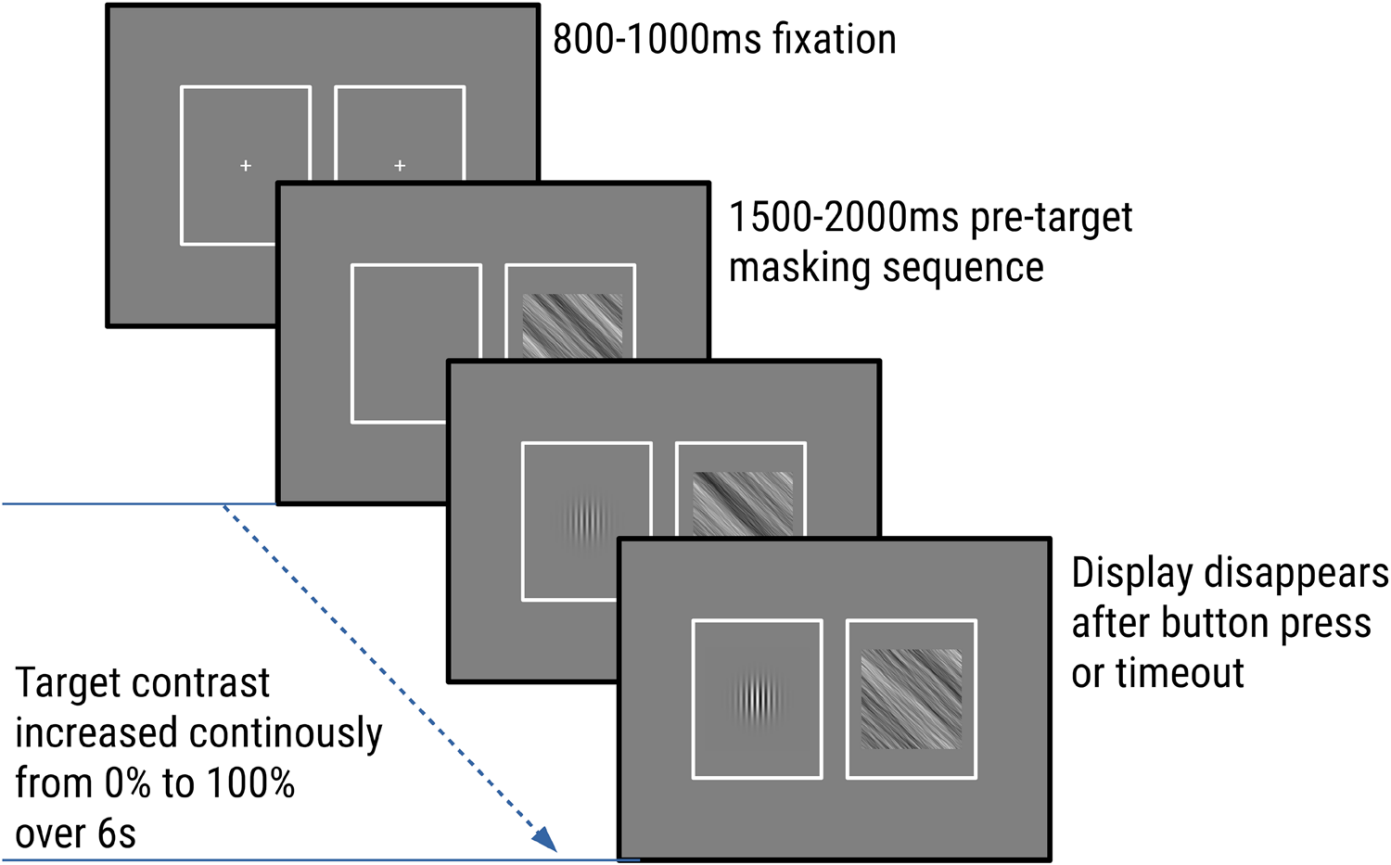
Experimental paradigm (breaking-CFS). The contrast of the stimulus images increased continuously from 0 to 100% over a period of 6 s. Participants were instructed to press the space button as quickly as possible when they saw the target.

A breaking-CFS paradigm was used, in which the masks were flashed to one eye at a predefined frequency, while the target stimulus was presented to the other eye, as illustrated in Fig. 2. Recently, Ding et al. suggested that eye dominance should be determined using a pretest of the same task that will be used in the experiment^44^. However, in order to be consistent with our previous studies, we continued to use the ABC test^45,46^ to determine the dominant eye. During each trial there were two static white frames (16.7° × 20.7°) surrounding the outer border of target stimuli and masks each presented on either side of the screen, such that both frames were visible only to their respective corresponding eye. In order to achieve good fusion between both frames, prior to the first trial of each block, the mirror setup as well as the distance between the two surrounding frames were adjusted for each participant.

The general paradigm was similar to previous studies^21,27,29^: participants started each trial with the press of a button. A central fixation cross extending 1° was then shown for a random period of 800–1000 ms. Subsequently, masks were flashed for a random period of 1500–2000 ms at a frequency of 6 Hz^21,27^ before the contrast of the target stimulus began to ramp up to the other eye. The contrast of the stimulus image increased from 0 to 100% linearly in 6 s (around 0.1% per display refresh with 8 bit precision), while the repetitive mask display to the dominant eye continued. Participants were instructed to press the space button on a keyboard as quickly as possible when they saw any part of the target image, but not before they could see it. Participants were informed that they would also experience trials without target (catch trials), on which they were not to give a response. After participants pressed the button, or after the trial timed out (6 s), the target image disappeared, and 3 more flashing masks were shown binocularly, to minimize possible effects of afterimages.

Every participant completed 5 blocks with 132 trials per block. Each block included 60 CFS-masked, target-present trials: four different mask orientations (− 45°, 0°, 45°, 90°), each presented with targets of 15 different angles of deviation (− 90°, − 45°, − 22.5°, − 11.25°, − 6.1°, − 3°, − 1.5°, 0°, 1.5°, 3°, 6.1°, 11.25°, 22.5°, 45°, 90° expressed relative to the mask orientation of the current trial). The + 90° and − 90° conditions were identical and were thus pooled for the statistical analysis (see “Statistical analysis” section). Further, there were 12 catch trials with balanced mask orientation (each of the 4 mask orientations occurred 3 times) and no target shown. Additionally, each block contained a total of 60 baseline trials similar to the CFS-masked trials, but without mask display. These trials were included to gauge any orientation dependence the target stimuli might exhibit independently of the masks. A total of 660 trials were thus collected from each participant, with the order of trials randomized within each block.

#### Participants

24 participants took part in the experiment (10 female, aged 20–27: mean = 23.8, sd = 1.4). All participants were students recruited from Yunnan University and were paid for their participation. The participants reported normal/corrected-to-normal vision and were naive to the purpose of the experiment. All experiments were approved by the ethics committee of Yunnan University and performed according to the principles expressed in the Declaration of Helsinki (2004). Written informed consent was obtained from all participants.

#### Statistical analysis

The median of the time of breakthrough (response time) of each mask type and each level of orientational deviation between masks and targets was computed per participant (due to the design of the experimental paradigm, the point of target break-through can be measured equivalently as either response time or contrast level). For comparisons, as indicated, the break-through time was analyzed by an ANOVA design for repeated measures with the factors mask orientation (absolute, 4 different angles) and orientational deviation (relative to the current mask, 14 different angles as + 90° and − 90° were pooled) as within-subject factors. We report the original degrees of freedom, corrected p-values and the Greenhouse–Geisser epsilon (εGG) where Mauchly’s test indicated significant deviations from sphericity. Statistical analysis was performed in IBM’s SPSS 26 as well as R 4.1.0^47^ using the rstatix package (v 0.7.0) and the ez package^48^.

### Results

One participant did not respond in a large number of trials (16.1%), which resulted in no valid trials in at least one condition. The participant was thus excluded from the analysis. On average across all conditions, the remaining 23 participants responded too early (before the stimulus was shown) on 0.6% of the trials (range 0–2.6%), or too late (no response before stimulus end) in 1.8% of the trials (range 0–6.5%). The catch trials were erroneously responded to on average in 2.0% of the corresponding trials (range 0–14.2%). Error trials were distributed homogenously across conditions, with no participant having less than 2 valid trials (out of 5 possible) in any condition. 99% of all participant/condition pairs had at least 4 valid trials and 91% of participant/condition pairs had all 5 valid trials.

**Figure 3:**
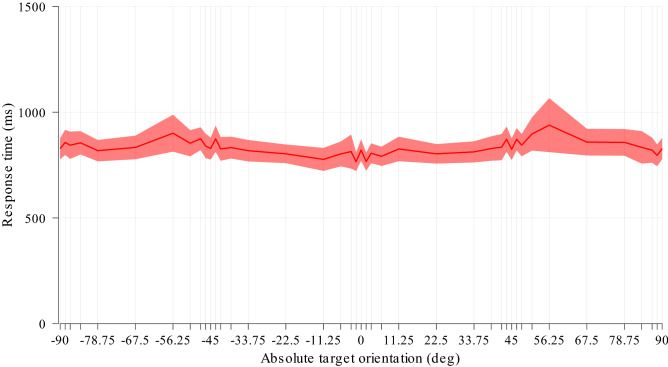
Experiment 1, Effects of target orientation in the baseline (no masking) condition. Response times of unmasked target stimuli sorted by absolute target orientation. Note that conditions at + 90° and − 90° are identical and have been duplicated for plotting. Mean and s.e.m. (23 participants).

**Figure 4:**
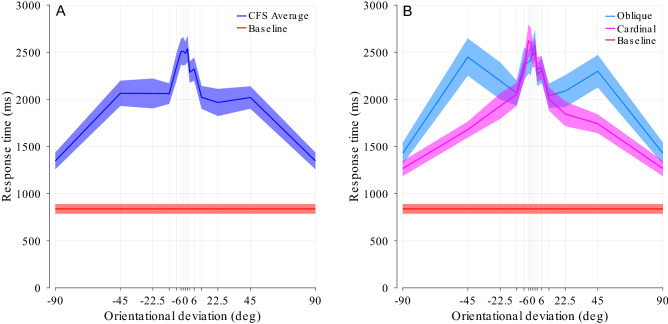
Experiment 1, main result. (**A**) Response times varied strongly with orientational deviation, peaking around the point of alignment between target and masks (0°). Blue line and shade represent mean and s.e.m (23 participants) of the pooled data in the CFS condition (with masking). Black line and shade represent mean and s.e.m. of the baseline condition (no masking). Note that conditions at + 90° and − 90° are identical and have been duplicated for plotting. (**B**) Data separated by oblique and cardinal mask orientations.

The performance in the baseline (no-masking) condition (Fig. 3) did depend on absolute target orientation (F(39,858) = 1.61, p = 0.011), with oblique target orientations exhibiting overall slower response times than cardinal orientations. However, the overall profile was very flat when compared to the CFS-masking condition (Fig. 4).

The main results of Experiment 1 can be seen in Fig. 4. Averaged across all target and mask orientations respectively, response time in the no-masking baseline condition was 838 ms (horizontal black line) compared to 2126 ms (254% of baseline) in the masking/CFS condition (paired t-test, t(22) = 12.37, p < 0.001). The response depended significantly on both mask orientation (F(3,66) = 3.59, p = 0.018, *η*^2^ = 0.012) as well as orientational deviation (F(13,286) = 19.14, p < 0.001, *η*^2^ = 0.144, εGG = 0.22), with a significant interaction (F(39,858) = 4.07, p < 0.001, *η*^2^ = 0.048). The shortest suppression was found at ± 90° deviation, with an average response time of 1348 ms (161% of baseline), while the longest suppression duration was found at near-zero deviation with a maximum of 2538 ms (303% of baseline) at 1.5° deviation, an increase of approximately 83% in absolute response time, or more than three times longer suppression (334% increase) when comparing to baseline. The difference between the values at 1.5 and 0° was not significant (paired t-test, t(22) = 0.64, p = 0.53), suggesting that the masking is highest when orientation of masks and target coincide.

When comparing between the cardinal and oblique mask orientations (Fig. 4B), the profiles of orientational deviation vs. mask orientation are markedly different. With the cardinal mask orientations, the fall-off in suppression duration when deviating from perfect alignment appears to be much narrower and reaches a higher maximum suppression duration than with the oblique mask orientations.

### Discussion

Experiment 1 showed that alignment between the orientational content of target and mask resulted in longer lasting suppression, while orthogonality between mask and target resulted in shorter suppression. The characteristics of the relation of the orientational alignment between mask and target appear to be similar to binocular rivalry, with suppression duration increasing in a relatively narrow peak around perfect alignment^37^. This general pattern differed strongly between oblique and cardinal mask orientations, which is consistent with accounts of the oblique effect in the human visual system^49^. The overall longer suppression duration when masks are shown at oblique angles (Fig. 4, dotted lines) and target orientations are not aligned may be explained by receptive fields pooling over a larger area^50^, allowing more of the comparatively larger masks to come into effect in these conditions, resulting in somewhat stronger suppression than the oblique effect would otherwise predict. With oblique masks, suppression times with cardinal targets (the ± 45° locations in Fig. 4B) are notably longer as well; we may hypothesize that this results from a tuning towards the oblique directions: the masks appear on the screen up to 2 s before the target starts to fade in, possibly allowing the visual system to tune its sensitivity more towards the oblique directions. In consequence, cardinal targets are then less privileged than they otherwise would be. In the same way, targets with ± 90° orientational deviation would then be within the oblique tuning, and orthogonal to the masks, and thus break through faster.

These findings provide strong evidence that an interaction between masks and targets in CFS does exist. They also show that CFS acts at least partially feature-specific rather than globally, as otherwise alignment of a single feature (orientation) should have no effect on suppression duration.

## Experiment 2: orientation with isoluminant colors

Experiment 1 resulted in clear evidence that mask-target interactions exit in CFS. However, in real life as well as many laboratory experiments, there may be and frequently are more than just one feature and therefore more than one dimension of similarity between masks and target. It is unclear in which way different features contained in both mask in target may interact, or if different features affect suppression independently. Extending from Experiment 1, we added a second controlled dimension to our stimuli.

In an isoluminant color domain, we designed stimuli to modulate along one of two orthogonal color axes (referred to as red–green and blue–yellow) in addition to the orientational tuning from Experiment 1. If similarity between target and masks are important for continuous flash suppression, we would predict that suppression duration will be longer when mask and target vary along the same color direction (both red–green or blue–yellow) than when they vary along different color directions (red–green and blue–yellow, or blue–yellow and red–green).

We also investigated whether the orientational alignment between stimuli always dominates the suppression duration, or if the alignment effect in the orientation domain will interact with the color domain—if mask and stimulus are designed along different (orthogonal) color axis rather than identical ones, will there still be an effect of orientational alignment, or will the orthogonality in the color domain allow the visual system to “circumvent” the orientational domain and allow the target stimulus to break through faster?

### Methods

#### Apparatus

The experimental apparatus was the same as in Experiment 1. The monitor was characterized with a X-Rite i1 pro 2. CIE1931 chromaticity coordinates and luminance of the monitor primaries were R = (0.6275, 0.3420, 18.8), G = (0.2870, 0.6108, 62.8), and B = (0.1483, 0.07232, 12.2). Gamma corrections without bit loss were applied based on the measured gamma curves of the monitor primaries.

#### Stimuli

**Figure 5:**
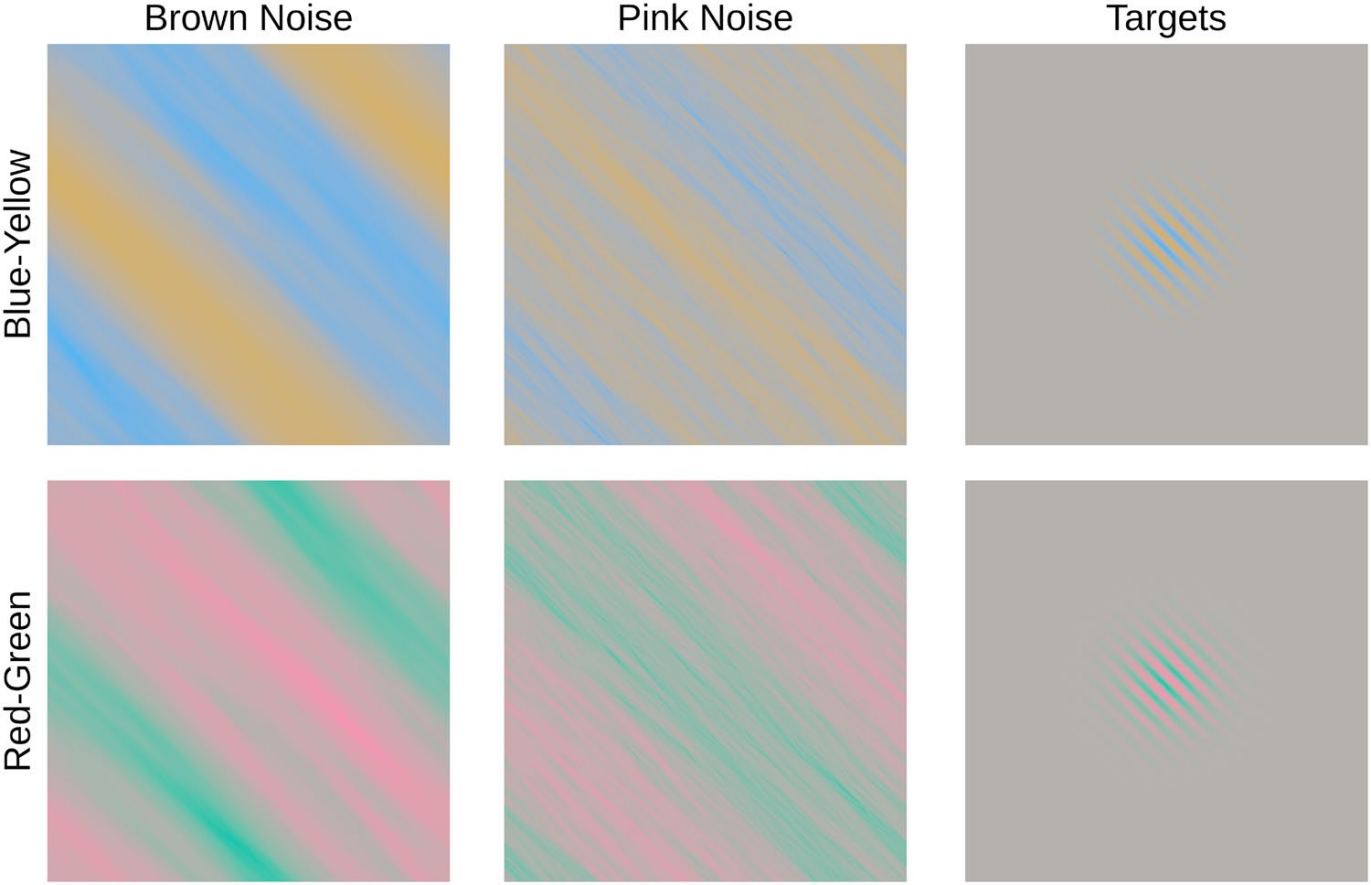
Isoluminant stimuli used in Experiment 2. Left columns: sample mask stimuli. Brown noise (1/f^2^) and Pink noise (1/f), both filtered with orientation Bandpass (8° fwhh). Right column: sample target stimuli. Gabor patches, oriented like the masks. Top row: Blue–Yellow color axis. Bottom row: Red–Green color axis. Note that visibility of the small illustration of stimuli may be compromised because of low-pass human chromatic contrast sensitivity^54^. The reader may improve visibility by increasing display size and/or moving closer to the display. Size of stimuli in the experiment was sufficient for them to be clearly visible.

Targets and Masks are illustrated in Fig. 5. Similarity between target and masks was varied in orientation (as in Experiment 1) as well as color. Orientation was manipulated similar to Experiment 1. Mask stimuli were designed in the same four orientations as in Experiment 1, and target stimuli were designed in 11 angles of deviation, identical to Experiment 1, but omitting the angles of 1.5° and 3° in either direction to limit the overall number of conditions.

The color variation defining the stimuli could either be the same for target and masks, or different (orthogonal). We represented colors in CIELAB color space to manipulate color contrast along orthogonal dimensions. CIELAB allowed us to approximately control perceived contrasts because it is a color appearance model that aims at compensating nonlinearities of color perception and approximates perceived color differences^51,52^. The adapting white-point was set to monitor white (xyY_1931_ = 0.2729, 0.2877, 93.7), and the grey background of all stimuli was achromatic (i.e., same chromaticities as white-point) at a lightness of L* = 70, which corresponds to a luminance of Y_1931_ = 38.2 cd/m^2^. The contrast of the Gabor patches was modulated either along a* (green–red) or along the orthogonal b* (blue–yellow) with a maximum CIELAB chroma of 40. This orthogonality allowed us to disentangle target-mask similarity in the color domain (same vs orthogonal) from the orientation domain. Luminance was held constant to avoid potential interactions between luminance and chromaticity.

Continuous flash suppression strongly depends on contrast^29^. While sensitivity to luminance contrast is band-pass with a peak at about 3–5 cpd, chromatic contrast sensitivity is low-pass reaching the maximum at about 0.5 cpd with little decrease towards lower frequencies^53^ (for a review see^54^). This implies that chromatic contrasts are more difficult to see for medium and high spatial frequencies compared to low spatial frequencies. For this reason, target stimuli were designed with a four times lower spatial frequency (1 cpd) than in Experiment 1 (4 cpd). We also added a second kind of noise mask. For one condition, we created masks with pink noise (1/f) as in Experiment 1, and for the additional condition, masks with brown noise (1/f^2^). Compared to pink noise, brown noise features precisely the same amplitude at the spatial frequency of the target (1 cpd) but allocates a larger amount of spectral energy on lower spatial frequencies and may therefore have higher potency as a mask when targeting the (low-pass) color system.

#### Procedure

Modified from Experiment 1, the experiment consisted of 2 masking conditions (Pink noise and Brown noise) with 8 subconditions each (2 different color axis in 4 orientations), paired with 22 target conditions (2 different color axis in 11 deviations each), with a sum of 176 condition pairs for each of the two masking conditions and an additional 176 trials without masking, resulting in a total of 528 target-present trials plus an additional 10 catch trials (target absent). As before, the + 90° and − 90° mask orientations were pooled for the statistical analysis. The order of all trials was randomized within each participant.

#### Participants

Fifteen individuals participated in the experiment (5 female, aged 19–35: mean = 23.3, SD = 4.3). All participants were students recruited from Yunnan University and were paid for their participation. The participants reported normal/corrected-to-normal vision and were naive to the purpose of the experiment.

### Statistical analysis

The median of the breakthrough contrast for each condition was computed per participant, and break-through contrast was analyzed by an ANOVA designs for repeated measures (see below). We report the original degrees of freedom, but corrected p-values and the Greenhouse–Geisser epsilon (εGG) where appropriate.

### Results

Of the 15 participants, one had to be removed due to a large number of no-response trials (8% of the total number of trials, leading to conditions without data), and a second one due to too many responses in catch trials (60%). The remaining participants responded to an average 3% of catch trials (range 0–10%), showed no response in 0.5% of trials (range 0–1.7%) and responded too early (before target onset) in 0.5% of trials (range 0–2.1%). Error trials were distributed homogenously; of the 352 CFS conditions, 302 (85.8%) had data from all 13 participants, further 48 (13.6%) had data from 12 of 13 participants, and 1 (0.03%) had data from 11 and 10 participants each No condition had data from less than 10 participants.

**Figure 6:**
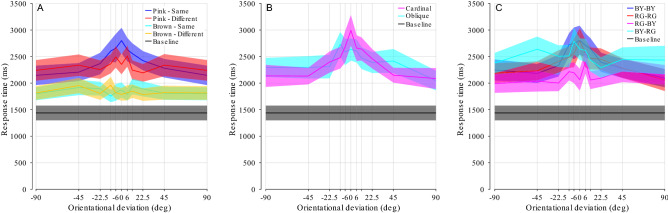
Results of Experiment 2. (**A**) The same-color pairings show a marked increase in suppression duration only for the pink noise masks, and only in the vicinity of zero orientational deviation. Mean and s.e.m. across 13 participants. (**B**) Data separated by cardinal and oblique orientations (Pink noise masks only). A trend similar to Experiment 1 emerges, with the differences between the mask orientations less pronounced. (**C**) Data separated by individual color pairings (Pink noise masks only). In the legend, target color is mentioned first, then mask color.

As in Experiment 1, we calculated median response times for each condition and participant to assess suppression effects. Figure 6 illustrates median response times averaged per stimulus condition. The comparison between the blue curve and the other curves suggests that pink noise produced the stronger suppression compared to brown noise, in particular when mask and target were designed along the same color axis (same-color pairings).

First, we conducted a one-way, repeated-measures ANOVA with the factor mask type (Pink noise, Brown noise or no masking) to test the statistical significance of continuous flash suppression in this experiment. The effect of mask type was significant (F(2,24) = 31.15, p < 0.001, *η*^2^ = 0.363, εGG = 0.66), indicating significant continuous flash suppression even though isoluminant stimuli were used. We then examined whether all masks produced suppression effects. For this purpose, we conducted separate t-tests comparing each color pairing and noise-type condition (colored curves in Fig. 6) with the baseline (grey curve in Fig. 6). All t-test were significant (min t = 3.79, max p < 0.005, Bonferroni-corrected), suggesting that all masks produced some level of continuous flash suppression.

We then concentrated on the difference between pink and brown noise, removing the control condition without masking from the analysis. We calculated a four-way ANOVA for repeated measures with the noise type (pink noise or brown noise, 2 levels), mask orientation (4 different angles: [− 45 0 45 90]), orientational deviation (10 angles: [± 90 − 45 − 22.5 − 11.25 − 6.1 0 6.1 11.25 22.5 45]) and color pairing (same vs. different color between mask and target, 2 levels) as within-subject factors. Mean response time in the baseline condition was 1436 ms, in the brown noise condition 1839 ms (128% of baseline) with different-color pairings and 1841 ms (128% of baseline) with same-color pairings, in the pink noise condition 2323 ms (162% of baseline) with different-color pairings and 2435 ms (170% of baseline) with same-color pairings. For the pink-noise, same-color pairings the minimum response time was 2146 ms (146% of baseline) and the maximum was 2801 ms (195% of baseline), resulting in a difference of 655 ms or a 92% increase in suppression duration after baseline subtraction.

All four main effects were significant: First, the effects of the similarity between target and mask orientation (F(9,108) = 4.39, p = 0.007, *η*^2^ = 0.268, εGG = 0.386), and second, the effects of mask orientation (F(3,36) = 3.27, p = 0.032, *η*^2^ = 0.214) were similar to those observed in Experiment 1 (cf. Fig. 4). The effect of color pairing between target and mask confirmed our prediction: suppression effects were generally stronger when target and mask were designed along the same color axis rather than different (F(1,12) = 5.50, p = 0.037, *η*^2^ = 0.314). This effect appears to be caused by the peak in suppression when mask and target orientations are aligned, which is strongly diminished if not entirely absent in different-color pairings. This indicates that same vs. different-color pairings achieve approximately the same overall suppression, however the increased suppression duration from orientational alignment between mask and target is mostly absent in different-color stimulus pairings. Fourth, and contrary to our prediction, pink noise yielded a higher suppression than brown noise masks (F(1,12) = 40.43, p < 0.001, *η*^2^ = 0.771).

All two-way interactions were also significant: First, we reproduced the interaction between mask orientation and orientational deviation (F(27,324) = 2.73, p < 0.001, *η*^2^ = 0.185) observed in Experiment 1 (cf. Fig. 6B). Second, the significant interaction between orientational deviation and color congruence (pairing) (F(9,108) = 3.08, p = 0.020, *η*^2^ = 0.204) indicated a higher effect of orientational deviation with same-color pairings than with different-color pairings (cf. Fig. 6A,C). This is in line with the idea that suppression effects are highest for masks that are most similar to targets. Third, the interaction between type of noise (pink vs brown) and orientational deviation (F(9,108) = 21.89, p = 0.005, *η*^2^ = 0.98, εGG = 0.37) showed that the effect of orientational deviation strongly occurred with pink noise masks, but barely with brown noise masks (different profile between red and blue, but similar profile for yellow and cyan curves in Fig. 6A). Fourth, the interaction between noise type and color congruence (F(1,12) = 5.96, p = 0.031, *η*^2^ = 0.332), implies that there was only an effect of color with pink, but barely any with brown noise masks (different height between red and blue, but similar height of yellow and cyan curves in Fig. 6A).

A three-way interaction further occurred between noise type, orientational deviation and color pairing (F(9,108) = 2.22, p = 0.026, *η*^2^ = 0.156). This interaction reflects the fact that the strongest suppression occurred when colors and orientations were very similar or the same for masks and targets, and when pink-noise was used for the mask (cf. center of blue curve in Fig. 6). There was also a three-way interaction between noise type, mask orientation and orientational deviation (F(27,324) = 1.52, p = 0.049, *η*^2^ = 0.113), showing that oblique orientations of masks had strongest suppression effects in the condition with pink noise and different-color stimulus pairings (local maxima of red curve in Fig. 6). There was no four-way interaction.

We focused on the pink noise condition to identify effects that are specific to colour variation (green–red vs blue-yellow, Fig. 6C). Suppression differed by orientational deviation (F(10,120) = 6.22, p = 0.02, *η*^2^ = 0.341, εGG = 0.34), target color (F(1,12) = 15.63, p = 0.002, *η*^2^ = 0.57), and mask color (F(1,12) = 26.73, p < 0.001, *η*^2^ = 0.69). Indeed, Fig. 6C shows that targets of the blue-yellow color axis appeared to have longer suppression duration than targets of the red–green color axis. A significant interaction between mask and target colors (F(10,120 = 5.1), p = 0.043, *η*^2^ = 0.30) shows that masks of the blue–yellow color axis resulted in the longest suppression with blue–yellow targets, but the shortest suppression with red–green targets. No significant interaction was found between target color and orientational deviation (F(10,120) = 1.22, p < 0.309, *η*^2^ = 0.092, εGG = 0.54), or mask color and orientational deviation (F(10,120) = 0.85, p = 0.512, *η*^2^ = 0.07, εGG = 0.43). The three-way interaction was significant (F(10,120) = 4.07, p = 0.002, *η*^2^ = 0.253, εGG = 0.53), showing that suppression duration ultimately depends on the combination of all three factors.

### Discussion

Experiment 2 delivered clear evidence that isoluminant conditions can elicit CFS. An emphasis of lower spatial frequencies in the masks (brown noise), intended to improve suppression when using isoluminant color-defined stimuli, did however not lead to an improvement in suppression duration, and in fact reduced suppression duration similar to what one might have expected with luminance-based masks^21,29^. It appears that pink noise is an optimal masking stimulus even under the increased perceptual demands placed on the visual system by the use of isoluminant stimuli. With Pink noise, in those conditions where both mask and target were designed along the same color axis, a strong effect of orientational alignment was found, similar to Experiment 1. This effect however was diminished when mask and target were of opposite color, suggesting that the effect of orientation on masking duration can be circumvented by the visual system by means of other, un-aligned feature dimensions. The suppression duration for orthogonal orientations was very similar between same color and different color pairings, indicating that basic suppression effectiveness with isoluminant stimuli is unaffected by differences in color, while the increased suppression duration from orientational alignment also requires alignment in the color domain. Looking at the two color axis independently, we find that blue-yellow masks resulted in the longest overall suppression with blue-yellow targets, but the shortest overall suppression with red–green targets, while red–green masks showed in comparison roughly similar suppression with both blue–yellow and red–green targets.

With the Brown noise masks, no effect of the orientational alignment became apparent, even in same-color conditions—even though overall significant suppression still occurred, as is evident from the comparison with the baseline condition.

In sum, both orientation and color similarity affected continuous flash suppression, but only with the pink-noise masks. The brown noise masks produced overall much weaker continuous flash suppression and barely varied as a function of orientation and color similarity (Fig. 6).

The weaker suppression by brown-noise masks is in line with previous observations for achromatic (luminance) stimuli^29^. If the congruence of spatial frequencies between targets and masks were key to the suppression effects, we expected similar effects of brown and pink noise for targets of 1 cpd. This, however, was not the case, suggesting a more complicated relationship between spatial frequencies of masks and targets. The precise nature of this relationship could be investigated by varying the spatial frequency of the target and the spatial frequency components of the noise mask to test what characteristics of spatial frequencies modulate continuous flash suppression.

Our results suggest that any kind of mask produces some level of continuous flash suppression, but that similarity may increase suppression effects. The role of similarity in continuous flash suppression is reminiscent of general forward and backward masking effects (i.e., without continuous flash). It has been suggested that forward and backward masking have similar effects to continuous flash suppression when tasks are designed to be comparable^55^.

## Experiment 3: orientation with RGB color

In Experiments 1 and 2, we presented evidence that CFS exhibits strong feature-based interactions between masks and targets. Orthogonality in one feature domain can apparently help overcome effects of alignment in another domain. However, in previous studies, masks (and targets) were rarely (if at all) designed along such carefully chosen independent feature domains. In most cases, such as the popular Mondrian-style masks, the contents of the masks are a random mixture of colors, luminance and orientations, although some regularities, such as a preference for horizontal and vertical orientations, may exist depending on the actual design of the masks. It is therefore interesting to see if mask/stimulus interactions still exist when feature dimensions are chosen without careful separation.

### Methods

We again chose spatial orientation and color as our independent feature domains, but without calibrating stimuli along isoluminant color axis. Instead, we chose the primary colors of the CRT display system (red, green and blue), as is frequently done when designing Mondrian masks^5,21,27^, even though we did not choose to mix color channels in order to maintain a feasible level of complexity, manifesting in a manageable number of conditions and trials.

#### Apparatus and stimuli

**Figure 7:**
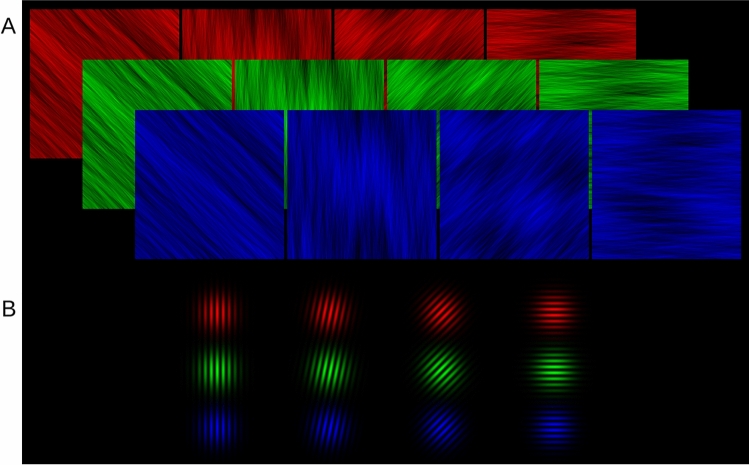
Stimuli in RGB colors as used in Experiment 3. (**A**) Sample of mask stimuli: Pink noise (1/f) filtered with orientation Bandpass (8° fwhh). (**B**) Sample of target stimuli: Gabor patches, oriented relative to the masks (only clockwise shown).

The experimental apparatus was the same as in Experiment 1. Similar to Experiment 2, we used pink noise filtered with a narrow orientation bandpass as masks and Gabor stimuli as targets. Stimuli in Experiment 3 were designed in the primary colors of the display system (RGB) with background luminance set to zero in order to best utilize both luminance and color contrast (Fig. 7).

### Procedure

Modified from Experiment 2, the experiment consisted of 3 different mask colors in 4 orientations each, paired with 3 different target colors in 7 deviations each (− 90°, − 45°, − 11.25°, 0°, 11.25°, 45°, 90°), for a sum of 252 target-present conditions with 2 repetitions each, resulting in a total of 504 target-present trials plus an additional 20 catch trials (target absent). As before, the + 90° and − 90° target deviations were pooled for the statistical analysis. The order of all trials was randomized within each participant.

### Participants

20 individuals participated in this experiment (7 female, mean age: 20–29, sd = 2.7). All participants were students recruited from Yunnan University and were paid for their participation. The participants reported normal/corrected-to-normal vision and were naive to the purpose of the experiment.

### Statistical analysis

Similar to the previous experiments, data was analyzed by an ANOVA design for repeated measures with mask orientation (4 levels), orientational deviation (6 levels) and color pairing (same vs. different color between mask and target, 2 levels) as within-subject factors. We report the original degrees of freedom, but corrected p-values and the Greenhouse–Geisser epsilon (_gg_ε) where appropriate.

### Results

Participants responded on average to 2.5% of the catch trials (range 0–10%), did not respond in 4.4% of trials (range 3.4–9.2%) and responded too early in 0.4% of trials (range 0–2.5%). Error trials were homogenously distributed. Of the 252 conditions, 152 (60.3%) had data from all 20 participants, 82 (32.5%) had data from 19 participants, 16 (6.3%) had data from 18 participants and 2 (0.8%) had data from 17 participants. No condition had data from less than 17 participants.

**Figure 8:**
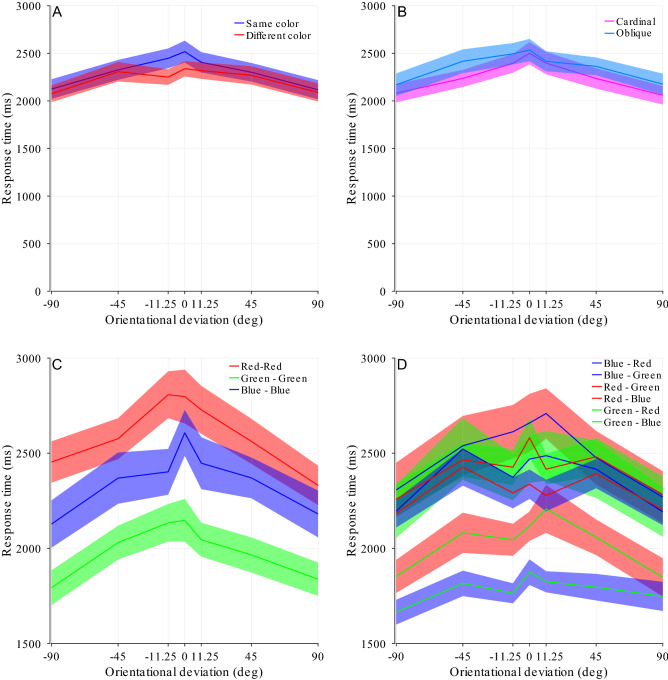
Results of Experiment 3: effect of different mask/stimulus color pairings. (**A**) The same-color pairings show a visible increase in suppression duration, but only in the vicinity of zero orientational deviation. Mean and s.e.m. across 20 participants. (**B**) Data from same-color pairings, separated by cardinal and oblique mask orientations. (**C**) Pink noise data, separated by color (same-color pairings only). A different scale was applied to facilitate data interpretation. (**D**) Pink noise data, separated by color (different-color pairings only). Target color is mentioned first in the legend and denoted by line color, mask color is noted second in the legend and denoted by shade color.

The results of Experiment 3 can be seen in Fig. 8. We found significant effects for the three main factors (mask orientation, F(3,57) = 8.29, p < 0.001, *η*^2^ = 0.012; orientational deviation, F(5,95) = 15.83, p < 0.001, *η*^2^ = 0.044, εGG = 0.46; color pairing, F(1,19) = 7.69, p = 0.012, *η*^2^ = 0.008), indicating that the basic orientational alignment effect as well as the effect of same vs. different color pairings found in the previous experiments also exist in Experiment 3 (Fig. 8A). The interaction between orientational deviation and color pairing (F(5,95) = 4.78, p < 0.001, *η*^2^ = 0.01) was also significant, indicating that orthogonality in the color domain can still help to overcome alignment in the orientation domain, even when color and luminance channels are confounded. Similar to Experiment 1, the interaction between mask orientation and orientational deviation (F(15,285) = 3.43, p = 0.003, *η*^2^ = 0.01, εGG = 0.41) was significant (Fig. 8B). No significant interaction was found between mask orientation and color pairing (F(3,57) = 1.46, p = 0.245, *η*^2^ = 0.001) or the interaction between all 3 factors (F(15,285) = 0.46, p = 0.861, *η*^2^ = 0.002, εGG = 0.46). When separating the data by target and mask colors, similar to Experiment 2, we find a significant effect of orientational deviation F(6,114) = 20.68, p < 0.001, *η*^2^ = 0.52, εGG = 0.33 as well as both target color (F(2,38) = 62.89, p < 0.001, *η*^2^ = 0.768, εGG = 0.72) and mask color (F(2,38) = 23.58, p < 0.001, *η*^2^ = 0.55, εGG = 0.77), as shown in Fig. 8C,D.

Target and mask color showed a significant interaction (F(4,76) = 4.37, p = 0.019, *η*^2^ = 0.187, εGG = 0.51) as is evident from Fig. 8D. While mask color interacted with orientational deviation (F(12,228) = 2.994, p = 0.001, *η*^2^ = 0.14), target color did not (F(12,228) = 1.56, p = 0.17, *η*^2^ = 0.76, εGG = 0.50). The three-way interaction between target color, mask color and deviation was marginally not significant (Mask × target × deviation F(24,456) = 1.77,* p* = 0.08,* η*^2^ = 0.085, εGG = 0.35).

### Discussion

From Experiment 3 we find that even when color and luminance are strongly confounded, orthogonality in an independent feature dimension (color) domain reduces the orientational alignment effect. The different primary color channels of CRT (and other) monitors are typically not of the same luminance; instead, they have been adjusted by the manufacturer to the standards commonly found in modern entertainment systems. This will certainly affect the staging of the different-color pairings (Fig. 8D) and may as well have an influence on the same-color pairings (Fig. 8C), so that the order of colors as it resulted in this study may depend somewhat on the monitor used. Despite that we can see that the different colors resulted in different suppression duration, even when the target was of the same color and thus of the same peak luminance as the masks. However, clean separation of color and luminance was not intended here. Rather, we intended to move one step in the direction of CFS masks commonly used in current studies, which typically vary in both color and luminance as deemed fit by the experimenters. We may not be able to make accurate claims of the degree of interaction between color, luminance and orientation from this experiment due to the lack of control inherent in the design of the stimuli; yet, we can reach the conclusion that the orientational alignment effect as well as the mitigation thereof by color orthogonality are likely to be found also in mask/stimulus pairings that were not expressly designed for the purpose of investigating mask-target interactions.

## Summary

We find that in continuous flash suppression, strong interactions can occur between the mask and the target. Targets that have features which are similar (aligned) to the features contained in the masks may be suppressed longer than targets that are very different (orthogonal), with an increase in suppression duration that can reach a factor of more than 3× relative to a no-masking baseline.

The strength of this effect of similarity in one feature dimension (orientation) may be reduced through orthogonality in another feature dimension (color).

In addition, previously little evidence existed regarding the possibility to elicit suppression in CFS with isoluminant stimuli^56^. Our data provides concrete evidence that continuous flash suppression does indeed work to a substantial extent even in the absence of luminance contrast in either mask or target stimuli.

The extent of the alignment effect on suppression duration varies with the overall orientation of the mask/stimulus-pair. When the masks are oriented along one of the cardinal axes, the alignment effect is both stronger and more narrow band than when the masks are oriented in one of the oblique directions. This may be explained by the oblique effect, according to which receptive fields preferring cardinal orientations are both more numerous and smaller than their oblique counterparts.

In Experiment 2, we employed two types of masks, pink noise and brown noise, that allocate their spectral energy to different amounts below and above the spatial frequency of the target. While pink noise is known to be the better CFS mask for conventional stimuli, we hypothesized that the human color system with its low-pass spatial frequency characteristics might be more receptive to masks with a higher amount of low spatial frequencies. This was however not the case; apparently, the rules that determine what optimally masks a stimulus are more complex than anticipated here and would warrant further scrutiny (future studies).

Similar effects can also be observed when stimuli are not carefully crafted along isoluminant color axis (Experiment 3), which indicates that these mask/target similarity effects may well and likely do occur in other experimental designs not intended for the same purpose as the experiments presented here. The degree of similarity between different types of targets and the masks used in CFS studies may thus bias the speed at which a target is detected.

## Conclusions

Varying degrees of similarity between the masks and different types of stimuli may lead to different suppression durations. Care needs to be taken not to interpret such differences in response times as expressions of differences in visual processing (in the absence of awareness) of the respective target stimuli; when the masks are not carefully designed to be equally similar to each tested target category, the differences may just originate simply from different degrees of similarity between the masks and the respective targets.

## Data Availability

The datasets analyzed during the current study are available from the corresponding author on reasonable request.
